# Changes in Doctor–Patient Relationships in China during COVID-19: A Text Mining Analysis

**DOI:** 10.3390/ijerph192013446

**Published:** 2022-10-18

**Authors:** Jiaxuan Li, Patrick Cheong-Iao Pang, Yundan Xiao, Dennis Wong

**Affiliations:** 1 Faculty of Applied Sciences, Macao Polytechnic University, Macao SAR 999078, China; 2 Research Institute of Forest Resource Information Techniques, Chinese Academy of Forestry, Beijing 100091, China; 3 Department of Computer Science, Stony Brook University, New York, NY 11794, USA

**Keywords:** doctor–patient relationship, doctor reviews, sentiment analysis, word frequency analysis, text mining, COVID-19

## Abstract

Doctor–patient relationships (DPRs) in China have been straining. With the emergence of the COVID-19 pandemic, the relationships and interactions between patients and doctors are changing. This study investigated how patients’ attitudes toward physicians changed during the pandemic and what factors were associated with these changes, leading to insights for improving management in the healthcare sector. This paper collected 58,600 comments regarding Chinese doctors from three regions from the online health platform Good Doctors Online (haodf.com, accessed on 13 October 2022). These comments were analyzed using text mining techniques, such as sentiment and word frequency analyses. The results showed improvements in DPRs after the pandemic, and the degree of improvement was related to the extent to which a location was affected. The findings also suggest that administrative services in the healthcare sector need further improvement. Based on these results, we summarize relevant recommendations at the end of this paper.

## 1. Introduction

The novel coronavirus disease (COVID-19) has been described as one of the greatest crises in global health, with over 580 million people infected and over 6.2 million deaths worldwide as of August 2022 [1]. As COVID-19 became a global pandemic, the progressive implementation of localized prevention policies, such as travel bans and mandatory mask-wearing, significantly changed people’s lifestyles [2]. Health professionals such as doctors and nurses played a significant role in controlling disease and taking care of patients, but because of the scarcity of medical resources, such as the lack of face masks and personal protective equipment, hospital beds, ventilators and other treatment equipment [3], there was a concomitant decline in attitudes towards doctors in some areas [4]. In general, the widespread of the virus has put enormous pressure on health systems in all countries [5].

In China, strained doctor–patient relationships (DPRs) have a long history, with research on the subject dating back to the 1990s [6]. In this history, China’s tensional DPRs have led to several deadly and vicious conflict cases. The Chinese government has also struggled to determine if China’s DPRs have changed or improved during all these years amid the introduction of public policies addressing this issue. In addition, medical professionals were more frequently present in people’s lives and public opinion during the pandemic [7]. Additional studies found that patients’ attitudes towards doctors do not remain static [8], and there are changes in DPRs reported after the emergence of COVID-19 [9].

To find a new way to assess the DPRs in China, we intend to conduct an exploratory study using patients’ online reviews. Online doctor reviews posted by patients are considered a tool to measure people’s attitudes and sentiments towards health professionals on a large scale [10]. While there are many research studies using online reviews to study public perceptions and comments [11], few of them leverage these data to analyze changes in patients’ attitudes toward physicians during COVID-19. There are even fewer that employ cross-sectional comparisons between areas based on reviews from different places. To this end, we aimed to explore the following research questions in this work:

(1) How did patients’ attitudes towards doctors change during the COVID-19 pandemic?

(2) Was the extent of the changes to patients’ attitudes towards doctors related to how the location was affected by the pandemic? 

(3) What management implications can be drawn from changes to such attitudes after COVID-19?

As an exploratory study, we used text mining techniques with sentiment analysis to analyze the polarity of sentiment in reviews and extract salient keywords from online doctor reviews. In addition, we compared the changes longitudinally before and during the pandemic in the same location as well as cross-sectional differences among three locations, i.e., Beijing, Shanghai and Hubei. We randomly collected 58,600 reviews of doctors from Beijing (12,000 each pre- and post-pandemic), Shanghai (12,000 each pre- and post-pandemic) and Hubei (5300 each pre- and post-pandemic). Then, we applied word frequency analysis as well as sentiment analysis techniques to integrate the results for the three locations. We also generated and compared the word cloud visualizations of the three locations.

Our results show that the number of negative reviews and highly negative reviews in Beijing decreased greatly from 348 to 259 (25.5%) and from 236 to 112 (52.5%), respectively. In Shanghai, the number of negative reviews and highly negative reviews decreased from 256 to 172 (32.8%) and from 134 to 46 (65.7%), respectively. The number of negative and highly negative reviews in Hubei decreased significantly from 79 to 58 (26.5%) and 37 to 11 (54.1%), respectively. Overall, Beijing saw the smallest reduction in negative reviews, followed by Hubei and finally Shanghai. In addition, we found that Beijing experienced service issues with registration before and after the pandemic. In Shanghai and Hubei, we found that online services were among the discussions of patient reviews.

In addition, this paper has several practical contributions. Our approach can provide hospitals and health departments with data-driven directions for improving their management. For instance, we believe Shanghai needs to strengthen its construction of online medical care, and Beijing needs to solve its issues related to the registration process. On the other hand, this work highlights the potential of applications that analyze patients’ reviews in the healthcare sector. Comparing data across locations allows us to monitor whether the level of healthcare is balanced and to measure whether local health policies are satisfactory.

## 2. Literature Review

In China, tensions in DPRs are a long-standing problem [12]. The earliest problem of doctor–patient conflict in modern China can be traced back to the late 1980s when the government implemented a set of ideas of “less money, more policy” to establish a medical system with “incentives, constraints, competition, and dynamism”. However, due to the serious asymmetry of medical information, there were only incentives but no constraints [13], which led to the earliest historical causes of doctor–patient conflicts in modern China. DPRs have become increasingly tense, and doctor–patient disputes are not just a simple medical problem but have evolved into a complex social problem [14]. There are many reasons for this problem, such as patients’ perceptions of a doctor’s professional status and the degree to which a doctor is replaceable within the profession [15]. The difficulty in addressing DPR issues lies in the need to use alternative methods to assess DPRs, and one study investigated DPRs by analyzing the content of internet message boards to construct a trust model [16].

In addition, the analysis of textual reports on the assessment of doctors allows the degree of professionalism and the quality of services to be determined [17], and patients’ feedback is influenced by the quality (competence, friendliness and honesty) of doctors, as well as online reputation such as online reviews and ratings [18]. As online medical communities become more appealing health channels, the frequency of physician-quality information updates and the quality of online services become increasingly critical for online physician–patient trust [19]. It is common knowledge that doctors’ educational and emotional support have a favorable impact on patient satisfaction. Moreover, emotional support has a greater impact on patient satisfaction than informational assistance. Furthermore, the severity of the patient’s condition improves the relationship between physician information, emotional support and patient satisfaction [20].

As COVID-19 spread rapidly to every corner of the world [21], it had an impact on the psyche of people. This was reflected in Twitter tweets about COVID-19, where there was a clear change in mood before and after the outbreak [22]. In addition, during the pandemic, people preferred to display their emotions through online messages [23], and the study found that fear dominated the early stages of the pandemic before shifting to anger and resentment. In terms of spatial distribution, public panic showed hierarchical and neighborhood diffusion, with highly assertive sentiments in the area where the pandemic had occurred, in economically developed areas and in areas surrounding the pandemic [24]. Furthermore, the demand for medical resources increased dramatically [25], leading to a sharp increase in psychological stress on doctors [26]. Some of the pandemic controlling measures during the pandemic led to changes in communication and trust between patients and doctors [27]. Studies have shown that the impact of preventive measures (such as wearing masks) on the lives of the public is significant, which can lead to an accumulation and increase in negative emotions [28]. In this context, it is crucial to investigate whether DPR has changed before and after COVID-19. Several researchers have investigated DPR with patients during the pandemic using questionnaires [29]. On the other hand, online information can be a valuable data source for studying DPRs [30]. In addition, the analysis of online data can complement traditional surveys to design and propose policies and interventions [31].

Online reviews are considered interpersonal communication. Semantic analysis based on reviews can reveal particular insights into public reactions to pandemics, and it is a proven qualitative research method [32]. This also applies to the health domain, including their experience analyzing access to services in an online environment [33]. For disease management, sentiment analysis in social media is important [34], and online reviews can also influence users’ decision-making processes [35]. People have been found to share information about the quality of their doctors [36], their attitudes [37], technical competence [38] and treatment effectiveness [39], as well as their experiences, on online health platforms. Although the number of studies in this field is relatively small, the current data are crucial to fight similar outbreaks in the future [40]. 

Data mining for thematic dynamics and sentiment trends can provide valuable knowledge about the opinions of the public [41]. Policymakers should consider these results and develop global health policies and surveillance systems by monitoring them [42]. Although online reviews about physicians have been studied in the literature, few studies have used online review data to determine similarities and differences in DPR after the COVID-19 outbreak. With the development of machine learning, information about geographic locations has been gradually used to study user-generated information [34]. Therefore, we hope to simultaneously study the sentiment analysis of multiple locations used to speculate whether the extent of the pandemic’s impact was related to changes in attitudes toward physicians.

## 3. Materials and Methods

This section describes our data collection and data preprocessing processes, followed by the methods of data analysis and the metrics used in this study.

### 3.1. Data Collection and Preprocessing

We gathered reviews from Good Doctors Online (haodf.com, accessed on 13 October 2022), a website where patients can submit feedback after visiting a doctor. With over 3 million daily visitors, 300,000 daily online consultations, and 580,000 registered doctors, Good Doctors Online is one of China’s most popular online health platforms [33]. While the site offers a variety of information (e.g., doctor’s name, age, condition name, location, affiliated hospital, etc.), this study only focused on reviews; therefore, other data was removed. There was no need to seek ethical approval because no personal information was involved in our study; only publicly available data was used.

We referred to the time in December 2019 when COVID-19 occurred in Wuhan, China [43], to further identify comments posted before and during the pandemic, and we compared the statistics from the year before to the year after. To make data categorization easier, we designated the comments made between January and December 2019 as “before the pandemic”. The comments posted between January and December 2020 were regarded as being made during the pandemic. From the online health platform, we randomly selected 58,600 reviews from doctors in Beijing (12,000 pre- and post-pandemic), Shanghai (12,000 pre- and post-pandemic) and Hubei (5300 pre- and post-pandemic). To determine the locations of patient reviews, since a user needed to specify a doctor on who they would like to comment, the city where the doctors worked was used in this case. In line with previous research [30,33], we removed symbols, links, abbreviations and stopwords from the raw data, as they had minimal relevance for our data analysis. In addition, short reviews (i.e., comments with less than five words) do not give sufficient lexical context for deciphering their meanings [44], and therefore they were excluded from our study.

### 3.2. Data Analysis

After extracting the data, we performed two text mining analyses to identify changes in DPRs and explore the potential causes of the changes separately. Sentiment analysis, which is a computational study of the sentiments surrounding an entity [45], was applied to measure the level of changes in DPRs. We used a third-party text sentiment analysis service known as the Baidu Cloud Sentiment Analysis System in this research. It is used by Baidu Nuomi (Baidu Cloud Computing Technology (Beijing) Co., Beijing, China), which is the top domestic life service software in China, and the system has also been used in other academic articles [46,47]. Two authors randomly assessed 200 comments analyzed by this third-party service and carefully evaluated the analysis results to confirm the accuracy of this service. The accuracy of sentiment classification achieved more than 90%, which is within our project’s tolerance [48].

The sentiment analysis results are expressed as decimals ranging from 0.0 to 1.0, which is a value that also represents the magnitude of the feeling. Text with negative emotions has a sentiment value of less than 0.5, and as negativity increases, the number approaches 0.0. Text with a sentiment value greater than 0.5, on the other hand, is regarded as positive. The positivity grows as the value approaches 1.0. We further separated the sentiment values into four levels in this study to obtain a finer granularity of sentiment, namely, highly positive (>0.9), positive (>0.5), negative (<0.5) and highly negative (<0.1). To perform statistical significance testing of the different sentiments, we used the normal distribution to approximate the binomial distribution and calculated the *p*-values.

On the other hand, word frequency analysis was used to identify words repeatedly used in positive and negative reviews by different patients and to identify the best-performing areas and the most common concerns expressed in the review comments. Researchers have utilized frequency analysis to leverage aspects of text corpora in order to investigate the context of the text [49], and we used this technique to understand salient themes in the comments. To convert sentences into word lists, we used PKUSEG [50], an open-source Chinese word segmentation library developed by Peking University. Furthermore, the Gensim library [51] was used to find double words or pairs of frequently used words. Because we only counted significant words in the study, we iteratively changed the stopwords used to delete some meaningless words after segmenting terms and recognizing double words. We counted the frequency of words after analyzing all of the data. To build word clouds, we chose 60 terms from each of the three locations before and during the pandemic (the top 30 words before and the top 30 words during the pandemic). These text analyses were based on Chinese; however, the results were translated into English by two bilingual authors.

We used several metrics to compare the healthcare systems in the three locations. A typical and functional healthcare system consists of interrelated building blocks: management and leadership, health information and technology, health workforce and health information managers, collectively known as health workers [52]. The WHO uses the doctor–patient ratio (DTPR) to assess regional health systems and has set a recommended value of 1:600 [52]. In order to comprehensively evaluating how a location was affected by the pandemic, we employed other different ratios: mortality showing the ratio of deaths, population-to-patient ratio (PTPR) showing the number of cases per 10,000 people and hospital-to-patient ratio (HTPR) showing the ratio of hospitals to the number of patient number.

## 4. Results

This section presents the results of sentiment analysis, cross-sectional analysis and word frequency analysis of the review data from the three locations.

### 4.1. Sentiment Analysis

In a total of 24,000 comments in the sample of Beijing, positive comments increased slightly by 0.8% from 11,652 before to 11,741 during the pandemic. For negative comments, there were 348 before the pandemic and 259 during the pandemic, showing a significant decrease (25% decrease; *p* < 0.001). Average sentiment values remained almost unchanged (0.9590 before the pandemic and 0.9595 during the pandemic) and increased by 0.1%. Very positive comments decreased slightly from 11,258 to 11,183 (−0.7%; *p* < 0.001). However, the number of strongly negative comments decreased significantly from 236 to 112, with a significant difference of 52.5% (*p* < 0.001). Table 1 presents descriptive data for Beijing on positive and negative comments.

In the total sample of 24,000 comments in Shanghai, positive comments increased slightly by 0.7%, from 11,744 to 11,828 during the pandemic. The number of negative comments decreased from 256 before the pandemic to 172 during the pandemic, which decreased by almost a third (−32.8%; *p* < 0.001). Additionally, there was a slight increase in the average sentiment value during the pandemic (from 0.9741 to 0.9789). Although it was not significant, there was also a slight increase (2.3%) in the number of highly positive comments during the pandemic compared to before. However, the number of highly negative comments during the pandemic was considerably less than before (−65.7% decrease; *p* < 0.001). Table 2 presents descriptive data for Shanghai on positive and negative comments.

For the sample with 10,600 comments in Hubei, there was a slight insignificant increase in the number of positive comments during the pandemic compared to the figure before the pandemic (0.3% increase). Conversely, the number of negative comments during the pandemic was considerably less than before the pandemic (26.5% decrease) (*p* < 0.001). There was essentially no change in the average sentiment value during the pandemic. Similarly, there were 0.3% more during the pandemic for highly positive comments. However, the number of highly negative comments during the pandemic was less than half of the number before the pandemic. Finally, Table 3 presents descriptive data for Hubei on positive and negative comments.

To understand how a place was affected by the pandemic, we used variables such as mortality, population and the number of doctors/hospitals to normalize changes in sentiment. We also included average data for the whole of China for reference. Then, we ranked the severity of the pandemic as follows: the smaller the numbers among the four assessment ratios, the less severe the pandemic is in the location. Table 4 shows the assessments of the severities of the pandemic. Among all places, Beijing had the lowest severity, with the smallest ratios of all variables except for PTPR. All four ratios of Shanghai were close to those of Beijing, and both Shanghai and Beijing were below the average of China. The Hubei region was more affected by the pandemic than Beijing and Shanghai, and it was worse than the national average. For PTPR, DTPR and HTPR, Hubei was 10 times higher than other places. For the changes in ASV, we found that Beijing, which was less impacted, e.g., had the lowest changes, followed by Hubei and then Shanghai. 

### 4.2. Word Frequency Analysis

**Figure 1:**
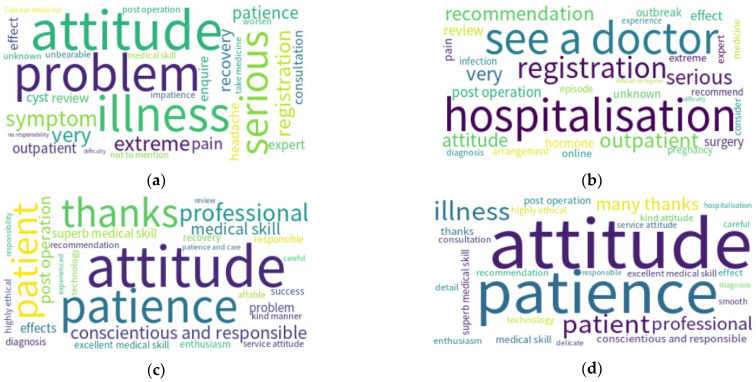
Doctor reviews from Beijing: (**a**) negative before the pandemic, (**b**) negative during the pandemic, (**c**) positive before the pandemic and (**d**) positive during the pandemic.

In this subsection, we compare the composition of words in positive/negative comments before and during the pandemic. Readers should note that these words were translated from Chinese to English in this subsection. In Table 5, we list the top 10 words used in negative comments in Beijing at different times. As shown in Table 5, the words used in Beijing before the pandemic included attitudes (e.g., attitude, patience/impatience and responsibility), and the magnitude of sicknesses (e.g., serious, recovery and worsen). On the other hand, the common words used in Beijing’s pandemic mainly concern access to health services, such as hospitalization, seeing a doctor and registration, with fewer words describing attitudes. Using side-by-side word cloud visualizations, we can further identify the differences before and during the pandemic (Figure 1).

In the results of positive word frequencies (Table 5), both periods (before and during the pandemic) in Beijing contained similar topics such as attitudes (e.g., “attitude”, “patience”, “conscientious” and “responsible”, etc.), medical skills and appreciation. The frequencies of these positive words were similar in both durations. Additionally, the top two words (“attitude” and “patience”) remained the same, while the third word changed from “thanks” to “patient”. As shown in the word cloud visualizations (Figure 1), the composition of positive words appeared similar before and during the pandemic. The dominant words included “attitude”, “patience”, “patient” and “professional”. The term technology exists in both lists before and during the pandemic.

In Table 6, we list the top 10 words used in negative comments in Shanghai at different times. As shown in Table 6, words used in Shanghai before the pandemic included attitudes, the severity of illness (e.g., “restoration”) and problems. On the other hand, common words used in the Shanghai pandemic mainly concerned descriptions of conditions and diagnoses, such as symptoms, post-operation and review, with fewer words describing attitudes. In contrast, the frequency of words regarding access to medical care (e.g., “online” and “registration”) during the pandemic was higher than before.

**Figure 2:**
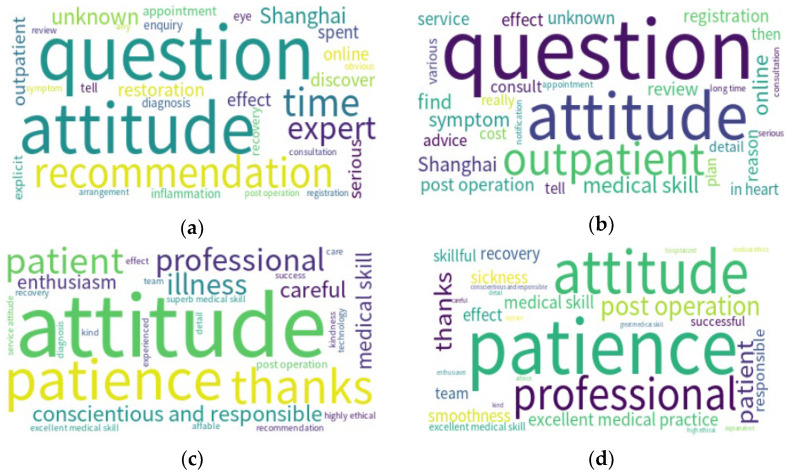
Doctor reviews from Shanghai: (**a**) negative before the pandemic, (**b**) negative during the pandemic, (**c**) positive before the pandemic and (**d**) positive during the pandemic.

For the results for positive word frequencies listed in Table 6, both periods in Shanghai contain similar themes such as attitude (e.g., attitude, patience, attentiveness, etc.), medical skills and appreciation. However, the frequency of words regarding medical skills was more prevalent before the pandemic. In addition, “thanks” and “professional” appeared in higher ranks than others. As the word cloud diagram shows (Figure 2), the composition of positive words appears to be similar before and during the pandemic. Dominant words included “attitude”, “patience”, “patience” and “professional”. The word “technology” was present in both lists before and during the pandemic.

In Table 7, we list the top 10 words used in negative comments in Hubei at different times. As shown in Table 7, words used in Hubei before the pandemic included attitudes, descriptions of diseases, treatment options (e.g., “anaemia” and “prescribing”) and “questions”. On the other hand, the common words used in the Hubei pandemic mainly described treatment effects. They commented on medical personnel (e.g., specialist registration, team and specialist), while fewer words were used to describe attitudes (attitude only ranked seventh).

**Figure 3:**
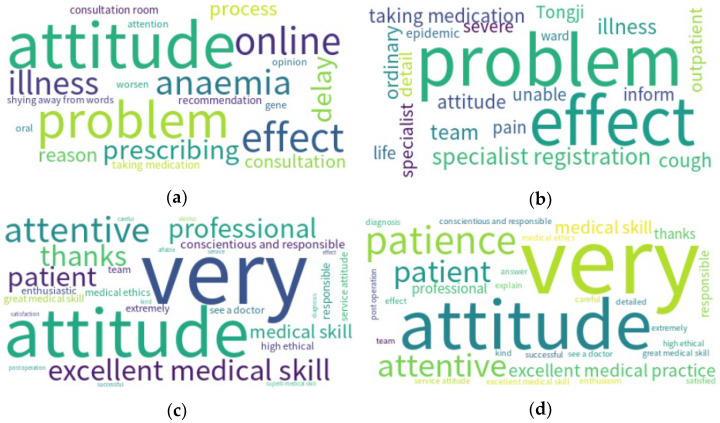
Doctors review from Hubei: (**a**) negative before the pandemic, (**b**) negative during the pandemic, (**c**) positive before the pandemic and (**d**) positive during the pandemic.

In the results for positive word frequency (Table 7), both periods in Hubei (before and during the pandemic) contain similar themes such as attitude (e.g., attitude, patience, attentiveness, etc.), medical skills and appreciation. However, the frequency of words concerning medical skills was higher before the pandemic. In addition, the top four words were all the same (very, attitude, patient and attentive), as shown in the word cloud diagram (Figure 3). The composition of positive words appeared to be similar before and during the pandemic. Dominant words included “attitude”, “patient” and “professional”. Many words appeared in both lists before and during the pandemic.

## 5. Discussion

In this section, we discuss the changes in DPRs implied by our results, the issues that emerged from hospital management and the methodological contributions developed in this paper.

### 5.1. Changes in DPRs

Based on the results, the number of negative reviews decreased significantly in all three locations, and the number and proportion of highly negative reviews decreased significantly. Both findings imply that patients’ attitudes and tolerance towards doctors or hospitals improved after the COVID-19 outbreak. At the same time, a cross-sectional comparison based on the three places found that Shanghai had the highest level of positive medical reviews in the overall favorable review comparison (Table 8). At the same time, highly negative ratings in Shanghai dropped the most during the pandemic (−65.7%), which may reflect an upturn in attitudes towards doctors in Shanghai during the pandemic. One possible explanation is that Shanghai was more affected by the pandemic. We assume that with the absence of a pandemic, people’s attitudes towards doctors would remain static from year to year because the number of negative reviews fluctuates very little every year based on experience. Therefore, if a location is less affected by an outbreak, fewer people would change their attitudes towards doctors. Conversely, the heavier the outbreak in an area, the more changes in attitudes towards doctors can be observed. These changes can be measured by the changes in positive and negative reviews from health consumers.

The above assumption can be applied to other locations in our studies. As shown in Table 8, the magnitude of change in Beijing during the pandemic was at a low level, and Beijing was the least damaged city by the pandemic. It had the least improvement in DPRs, which is in line with our argument. However, Hubei was the place most damaged by the pandemic, but the improvement of DPRs was not as great as that of Shanghai. We further argue that this is because Hubei had experienced a severe outbreak that was even worse than China’s average. Ji et al. [39] suggested that patients’ prognoses had been badly affected in Hubei due to the severe shortage of healthcare resources when COVID-19 was first detected there, and this also led to a smaller change of DPRs during the pandemic. 

Nevertheless, we can see that the extent of the impact of the pandemic and the improvement in attitudes towards doctors are positively correlated in general. There was an increase in empathy for doctors as the news and social opinion in China played up doctors’ hard work and hardship. Furthermore, the level of positive comment sentiment remained stable across the dataset before and during the pandemic, the composition of the words used was similar and the proportion of positive comments was similar. This means that patients’ satisfaction was not altered by the pandemic. As demonstrated in the results, what was changed by the pandemic was empathy and tolerance, as the number of negative emotions decreased. Similarly, another study from an Asian country (India) regarding text sentiment analysis of COVID-19 showed that negative reviews were much fewer than positive reviews [34]. Factors including expertise (e.g., medical skills), appreciation and communication skills (e.g., attitude) remained the dominant factors leading to positive feedback during the pandemic, as shown in the word clouds. These keywords continued to appear in the list both before and during the pandemic, suggesting patients’ awareness of smart health and other intelligent medical technologies. These are potential key directions for continued improvement in DPRs after this pandemic.

### 5.2. Issues of Hospital Management

As shown in the word clouds, before the outbreak, many of the terms in the negative comments were mainly directed at physicians, e.g., “attitude”, “problem”, “question”, etc. It was similar in all three locations and in line with the literature review highlighting that the DPRs in China were tense. In contrast, in the aftermath of a pandemic, high-frequency terms became mostly associated with administrative problems at the time of the outbreak. Although there were also words targeting doctors or attitudes, they all decreased accordingly, which reflects that the outbreak led to increased consumers’ tolerance of doctors. On the other hand, we found a shift in vocabulary after the outbreak, and words related to the administration and workflows began to appear (e.g., registration). Therefore, we speculate that COVID-19 led to a shortage of resources for hospital care and exposed many administrative problems in the health system, such as difficulties in registration and hospitalization, which contributed to the poor patient experience. The use of information technology and health information systems can assist with tasks such as remote triage or organizing hospital visits online. Patient-oriented digital healthcare can provide insights to alleviate such administrative problems. Finally, we compared the vocabularies of the three locations. We found that positive vocabulary remained consistent across different places, but some differences could be found in the negative vocabulary. During the pandemic, the number of the word “registered” and its ranking both increased.

The word “registration” in Chinese refers to a series of administrative or management problems in hospitals, such as difficulties in registration and complicated registration methods. This represents that the problem of registration became worse during the pandemic. Secondly, the word lists of Shanghai did not have the word “online” before the pandemic but contained “online” during the pandemic, which may be related to the inadequate online medical consultation or online registration system in Shanghai after the pandemic. In contrast, Hubei had “online” in its negative vocabulary before the pandemic but not after the pandemic. We argue that this may reflect the relatively good online system developed in Hubei during the pandemic, and it was favored by the public. Wuhan, Hubei province, launched the country’s first Internet health platform, “Your Health”, in the first half of 2021 [53]. This platform supports the entire process of medical services, such as online consultation, online purchase of medicine, online medical insurance payment and home delivery. It provides high-quality online health services for patients. Therefore, this confirms our explanation for the disappearance of the word “online” during the pandemic in Hubei.

### 5.3. Methodological Contributions

As an experiment, we used data from online patient reports to assess patients’ emotions based on the assessment of the severity of a pandemic. While many papers investigating patient sentiment and attitudes during COVID-19 used a retrospective approach (in questionnaires or interviews), our work applies a data-driven approach to data before and during COVID-19, respectively. Furthermore, we used the data to find commonalities and differences based on a comparison of the three locations to determine the likelihood of influencing patient mood and identify the strengths and weaknesses of inter-regional healthcare systems. It can help the government to understand whether the distribution of interregional medical information is reasonable. For example, Article 8 of the Interim Regulations on Quality Management of Outpatient Clinics in Medical Institutions, issued by the China Health Care Commission in June 2022 [54], states that “medical institutions should reasonably allocate human resources for outpatient clinics according to geographical or seasonal characteristics”. Our research can help the country to deploy healthcare in regions across the country. On the other hand, it can be one of the evidence-based approaches to evaluate the usefulness of healthcare policies or healthcare reforms. For example, negative reviews in Shanghai during the pandemic appeared with the keyword “online”, suggesting online aspects were a pain point that people complained about pre-pandemic. With the upgraded and reformed healthcare system in the Shanghai area afterwards, we can observe that the frequency of the negative word “online” decreased, which implied that this reform was effective.

### 5.4. Limitations

There are several limitations to this study. First, the reviews were self-reported by the patients and only reflected patients’ points of view. Additionally, it is possible that patients cannot fully express all their feelings in words. Secondly, the distribution of positive and negative reviews was skewed, as the website listed far more positive reviews than negative ones. Thirdly, our study is limited to three places in China, and future studies should include data from different geographical locations.

## 6. Conclusions

We presented a study comparing patients’ attitudes in three Chinese locations before and during the COVID-19 pandemic. The results of the text mining showed a decrease in the number of negative comments in all three places. Therefore, we conclude that patients’ attitudes towards doctors changed, and the change was related to the extent to which the region was affected by the pandemic. In addition, word frequency and word cloud analyses found a consistent shift in negative terms from targeting doctors to raising issues with hospitals or medical services before and after the pandemic. Digital health technologies, smart hospitals and policy changes can contribute to mitigating these issues in this ongoing COVID-19 pandemic.

## Figures and Tables

**Table 1:** The Numbers of Comments with Different Sentiments (Beijing).

	Before Pandemic	During Pandemic	Difference	*p*-Values
Total	12,000	12,000	---	---
Number of Positive	11,652	11,741	+0.8%	0.999
Number of Negative	348	259	−25.5%	<0.001 *
Average Sentiment Value	0.9590	0.9595	+0.1%	0.501
Number of Highly Positive	11,258	11,183	>0.7%	<0.001 *
Number of Highly Negative	236	112	>52.5%	<0.001 *

* *p* < 0.05.

**Table 2:** The Numbers of Comments with Different Sentiments (Shanghai).

	Before Pandemic	During Pandemic	Difference	*p*-Values
Total	12,000	12,000	---	---
Number of Positive	11,744	11,828	+0.7%	0.999
Number of Negative	256	172	−32.8%	<0.001 *
Average Sentiment Value	0.9741	0.9787	+0.5%	0.487
Number of Highly Positive	11,403	11,671	+2.3%	0.999
Number of Highly Negative	134	46	−65.7%	<0.001 *

* *p* < 0.05.

**Table 3:** The Numbers of Comments with Different Sentiments (Hubei).

	Before Pandemic	During Pandemic	Difference	*p*-Values
Total	5300	5300	---	---
Number of Positive	5221	5237	+0.3%	0.965
Number of Negative	79	58	−26.5%	<0.001 *
Average Sentiment Value	0.9726	0.9751	+0.3%	0.506
Number of Highly Positive	4996	5011	+0.3%	0.812
Number of Highly Negative	37	17	−54.1%	<0.001 *

* *p* < 0.05.

**Table 4:** The Ratios of Four Measures of Pandemic Severity.

	Cases	Mortality	PTPR	DTPR	HTPR	ASV Difference	Severity Ranking
China	87,071	0.7%	6.22 × 10^−^^5^	1.93 × 10^−2^	2.51 × 10^−1^	---	(For Reference)
Hubei	68,149	2.9%	1.18 × 10^−^^3^	4.48 × 10^−^^1^	1.92	+0.3%	1
Shanghai	1511	0.46%	6.07 × 10^−^^5^	7.95 × 10^−^^2^	2.56 × 10^−^^1^	+0.5%	2
Beijing	982	0.45%	7.95 × 10^−^^5^	7.95 × 10^−3^	8.66 × 10^−2^	+0.1%	3

ASV: Average sentiment value; DTPR: Doctor-to-patient ratio; PTPR: Population-to-patient ratio; HTPR: Hospital-to-patient ratio.

**Table 5:** Frequently Used Words in Comments (Beijing).

Positive Comments (Beijing)		Negative Comments (Beijing)	
Before Pandemic	During Pandemic	Before Pandemic	During Pandemic
1. attitude (1589 times)	1. attitude (1589 times)	1. attitude (59 times)	1. hospitalization (29 times)
2. patience (1258 times)	2. patience (1258 times)	2. problem (48 times)	2. see a doctor (28 times)
3. thanks (870 times)	3. thanks (870 times)	3. illness (45 times)	3. registration (28 times)
4. excellent medical skills (741 times)	4. excellent medical skills (741 times)	4. serious (27 times)	4. outpatient (19 times)
5. patient (725 times)	5. patient (725 times)	5. symptom (24 times)	5. serious (19 times)
6. professional (698 times)	6. professional (698 times)	6. very (23 times)	6. recommendation (16 times)
7. conscientious and responsible (652 times)	7. conscientious and responsible (652 times)	7. extreme (23 times)	7. very (16 times)
8. careful (639 times)	8. careful (639 times)	8. registration (18 times)	8. attitude (15 times)
9. post operation (532 times)	9. post operation (532 times)	9. patience (17 times)	9. post operation (15 times)
10. medical skill (512 times)	10. medical skill (512 times)	10. recovery (17 times)	10. review (15 times)

**Table 6:** Frequently Used Words in Comments (Shanghai).

Positive Comments (Shanghai)		Negative Comments (Shanghai)	
Before Pandemic	During Pandemic	Before Pandemic	During Pandemic
1. attitude (1740 times)	1. patience (1755 times)	1. question (44 times)	1. question (33 times)
2. patience (1466 times)	2. attitude (1590 times)	2. attitude (38 times)	2. attitude (18 times)
3. thanks (933 times)	3. professional (975 times)	3. recommendation (28 times)	3. outpatient (16 times)
4. excellent medical skills (800 times)	4. post-operation (925 times)	4. time (27 times)	4. medical skill (14 times)
5. patient (755 times)	5. patient (834 times)	5. expert (23 times)	5. online (13 times)
6. professional (719 times)	6. thanks (821 times)	6. Shanghai (22 times)	6. find (12 times)
7.careful (659 times)	7. excellent medical practice (820 times)	7. unknown (22 times)	7. symptom (12 times)
8. illness (647 times)	8. medical skill (789 times)	8. restoration (20 times)	8. Shanghai (12 times)
9. conscientious and responsible (568 times)	9.careful (749 times)	9. outpatient (19 times)	9. post-operation (12 times)
10. careful (557 times)	10. recovery (660 times)	10. discover (19 times)	10. review (12 times)

**Table 7:** Frequently Used Words in Comments (Hubei).

Positive Comments (Hubei)		Negative Comments (Hubei)	
Before Pandemic	During Pandemic	Before Pandemic	During Pandemic
1. very (1292 times)	1. very (1361 times)	1. attitude (22 times)	1. problem (9 times)
2. attitude (727 times)	2. attitude (663 times)	2. problem (13 times)	2. effect (8 times)
3. patient (589 times)	3. patience (655 times)	3. effect (8 times)	3. specialist registration (7 times)
4. attentive (463 times)	4. attentive (476 times)	4. online (7 times)	4. illness (6 times)
5. excellent medical skill (395 times)	5. patient (430 times)	5. anemia (7 times)	5. team (6 times)
6. thanks (384 times)	6. excellent medical practice (363 times)	6. illness (6 times)	6. taking medication (5 times)
7. patient (350 times)	7. medical skill (361 times)	7. prescribing (6 times)	7. attitude (5 times)
8. professional (332 times)	8. professional (359 times)	8. delay (6 times)	8. cough (5 times)
9. medical skill (329 times)	9. thanks (347 times)	9. process (5 times)	9. ordinary (5 times)
10. conscientious and responsible (299 times)	10. responsible (312 times)	10. reason (5 times)	10. inform (4 times)

**Table 8:** Sentiment Analysis Summary for Beijing, Shanghai and Hubei.

	Beijing	Shanghai	Hubei
Total	12,000	12,000	5300
Number of Negative (Before Pandemic)	348	256	79
Number of Negative (During Pandemic)	259	172	58
Number of Negative Difference *p*-values about Number of Negative	−25.5% <0.001 *	−32.8% <0.001 *	−26.5% <0.001 *
Average Sentiment Value (Before Pandemic)	0.9590	0.9741	0.9726
Average Sentiment Value (During Pandemic)	0.9595	0.9787	0.9751
Average Sentiment Value Difference *p*-values about Average Sentiment Value Difference	+0.1% 0.501	+0.5% 0.487	+0.3% 0.506
Number of Highly Negative (Before Pandemic)	236	134	37
Number of Highly Negative (During Pandemic)	112	46	17
Number of Highly Negative Difference *p*-values about Number of Highly Negative	−52.5% <0.001 *	−65.7% <0.001 *	−54.1% <0.001 *

* *p* < 0.05.

## Data Availability

Data is available upon request.
